# Serum Dickkopf-1 expression level positively correlates with a poor prognosis in breast cancer

**DOI:** 10.1186/s13000-014-0161-4

**Published:** 2014-08-13

**Authors:** Shao-jie Zhou, Shou-rong Zhuo, Xiao-qing Yang, Chun-xin Qin, Zi-liang Wang

**Affiliations:** Department of Breast and Thyroid Surgery, Weihai Municipal Hospital, 70 HePing Road, Weihai, Shandong 264200 China

**Keywords:** Dickkopf-1, Serum, Breast cancer, Prognosis, Survival rate

## Abstract

**Background:**

The different expression level of Dickkopf-1 (DKK-1) in different cancers shows that the function of DKK-1 depends on the histological type of the cancer cells and the tissue microenvironment. To our knowledge, the serum expression level of DKK-1 in breast cancer is little known.

**Methods:**

Blood samples from 125 consecutive patients diagnosed with breast cancer and 53 control subjects from March 2008 to August 2013 were investigated. Serum DKK-1 expression levels were measured by enzyme-linked immunosorbent assay (ELISA). The overall survival (OS) and relapse-free survival (RFS) analyzed by log-rank test, and survival curves were plotted according to Kaplan–Meier.

**Results:**

The mean serum level of DKK-1 in patients with breast cancer was 4.99 ± 1.50 ng/mL, and was significantly higher than that in healthy individuals (1.88 ± 0.81 ng/mL, *P* < 0.001). DKK-1 level correlated significantly with TNM stage (*P* = 0.009), tumor grade (*P* = 0.02), lymph node metastasis (*P* = 0.001), and expression of HER2 (*P* = 0.002). The DKK-1 expression level was classified as high or low in relation to the median value, and patients with breast cancer (n = 125) were divided into a high expression group (n = 63) and a low expression group (n = 62). The Kaplan-Meier method for survival analysis showed that the patients with a high serum DKK-1 level had a poorer OS (48.7% vs. 81.3%, p = 0.01) and RFS (24.3% vs. 71.6%, p = 0.003) than those with a low expression level. The multivariate Cox regression analysis indicated that serum DKK-1 level was independent prognostic factors for OS and RFS.

**Conclusions:**

Serum DKK-1 level can be used as a noninvasive biomarker for the prognosis of breast cancer.

**Virtual slides:**

The virtual slide(s) for this article can be found here: http://www.diagnosticpathology.diagnomx.eu/vs/13000_2014_161

## Background

Breast cancer is the most common malignancy and is the leading cause of cancer death in females worldwide, accounting for 23% of the total cancer cases and 14% of the total cancer deaths [1]. Invasion and metastasis are the major features of malignant tumors and result in poor prognosis of patients. The invasive abilities of cancer cells are the critical parameters of the metastatic cascade [2]. Therefore, better understanding of the prognosis and metastasis associated factors and the underlying mechanisms would help to improve patients’ prognosis. Although some pathological factors, including estrogen receptor (ER), progesterone receptor (PR) and c-erbB-2 (HER2), have been widely used as a reference in clinical diagnosis and treatment, their prognostic value for breast cancer still has certain limitations. Therefore, it is important to identify reliable prognostic markers in clinical practice for the treatment of breast cancer [3-5].

Dickkopf-1 (DKK-1), a secreted protein, is known as a negative regulator of the Wnt signaling pathway [6]. DKK-1 binds to lipoprotein receptor-related protein-5/6 (LRP5/6) and blocks interaction with Wnt-1, resulting in β-catenin degradation and retardation of proliferation [7-9]. The expression and roles of DKK-1 are different in various cancers, current studies have reported that overexpression of DKK-1 is found in many malignant tumors, including lung cancer, esophageal carcinomas, cervical cancer, and hepatocellular carcinoma (HCC), indicating a potential oncogenic function of DKK-1 [10-13]. However, paradoxically, the expression of DKK-1 was down-regulated significantly in human colon cancer, gastric cancer and melanoma [14-16], suggesting that the function of DKK-1 may be different in different types of cancers. In spite of these studies, there little has been reported on the significance of DKK-1 expression in breast cancer progression and prognosis.

In the present study, serum expression level of DKK-1 of 125 patients diagnosed with breast cancer was examined using enzyme-linked immunosorbent assay (ELISA), and the correlations between serum DKK-1 expression and clinicapathological factors were explored. Furthermore, the prognostic role of DKK-1 in breast cancer was evaluated using Cox regression and Kaplan-Meier analysis.

## Methods

### Patient, and blood samples

Blood samples from 125 consecutive patients diagnosed with breast cancer who underwent surgery at the Department of Breast and Thyroid Surgery, Weihai Municipal Hospital from March 2008 to August 2013 were investigated. 53 control subjects were randomly selected among individuals receiving health examinations at the Health Examination Center of Weihai Municipal Hospital, any of these subjects who had a history of cancer were excluded from the study. Blood samples were collected from the patients at the time of diagnosis, before surgery. The demographic and pathological data, including age, gender, and the tumor stage, were obtained by a review of the patients’ medical records. The protocol for this study was approved by the ethics committee of Weihai Municipal Hospital, and written informed consent was obtained from each participant. A structured questionnaire was administered by well-trained interviewers to collect information on demographic and anthropometric characteristics of the enrolled subjects. Venous blood samples were collected into anticoagulant-free tubes and centrifuged to obtain serum samples, which were stored at −80°C until they were assayed.

### Enzyme-linked immunosorbent assay

Serum DKK-1 expression levels were measured by enzyme-linked immunosorbent assay (ELISA) with immunoassay kit (Miltenyi, Germany) according to the manufacturer’s directions. The optical density (OD) at 450 nm was determined. The standard curves were established with OD450 as Y axle and the concentration of standard substance as X axle. The level of protein was obtained through standard curve. Results are reported as concentration of DKK-1 ng/ml in samples.

### Statistical analysis

Numerical variables were recorded as means ± SD and analyzed by independent t-tests. Categorical variables were presented as rates and analyzed by using the chi-square test or Fisher’s exact test. The relapse-free survival (RFS) was defined as the interval between the operation and the date that tumor recurrence or metastasis were diagnosed. Overall survival (OS) was defined as the interval between the operation and the date of death of the patient. The OS and RFS analyzed by log-rank test, and survival curves were plotted according to Kaplan–Meier. Univariate Cox regression was performed on each clinical covariate to examine its influence on patient survival. Final multivariate models were based on step-wise addition. P-values <0.05 were considered as statistically significant. Statistical analyses were performed using SPSS 13.0 soft-ware (Chicago, Ill., USA) and GraphPad Prism 5 (GraphPad Software Inc., CA, USA).

## Results

### DKK-1 levels in serum of breast cancer

**Figure 1:**
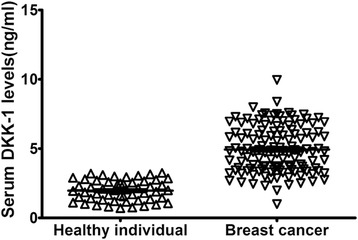
**DKK-1 levels in serum of breast cancer and healthy individuals.** The mean serum level of DKK-1 in patients with breast cancer was 4.99 ± 1.50 ng/mL, and was significantly higher than that in healthy individuals (1.88 ± 0.81 ng/mL, p < 0.001).

The DKK-1 levels in serum from patients with breast cancer (n = 125), and from healthy individuals (n = 53) were detected by ELISA. The mean serum level of DKK-1 in patients with breast cancer was 4.99 ± 1.50 ng/mL, and was significantly higher than that in healthy individuals (1.88 ± 0.81 ng/mL, *P* < 0.001, shown in Figure 1).

### Serum DKK-1 levels and clinicopathological characteristics

The DKK-1 expression level was classified as high or low in relation to the median value, and patients with breast cancer (n = 125) were divided into a high expression group (n = 63) and a low expression group (n = 62). The relationships between serum DKK-1 levels and clinicopathological characteristics of patients with breast cancer were analyzed (shown in Table 1). Serum DKK-1 levels correlated significantly with TNM stage (*P* = 0.009), tumor grade (*P* = 0.02), lymph node metastasis (*P* = 0.001), and expression of HER2 (*P* = 0.002). There was no significant correlation of serum DKK-1 levels with age (*P* = 0.61), histological type (*P* = 0.11), expression of ER (*P* = 0.09), expression of PR (*P* = 0.12), family history of breast cancer (*P* = 0.07), radiation therapy (*P* = 0.39), chemotherapy (*P* = 0.31), and menopausal status (*P* = 0.45).

**Table 1:** **The serum levels of DKK-1 according to clinicopathologic characteristics of patients with breast cancer**

		**DKK-1 expression**		
**Variables**	**Cases (n)**	**High**	**Low**	**p value**
Age (years)				
<50	59	27	32	0.61
≥ 50	66	36	30	
TNM stage				
I + II	71	26	45	0.009*
III	54	37	17	
Grade				
G1-G2	67	24	43	0.02*
G3	58	39	19	
Histological type				
Ductal	88	49	39	0.11
Others	37	14	23	
Lymph node metastasis				
Yes	46	36	10	0.001*
No	79	27	52	
ER				
Negative	59	33	26	0.09
Positive	66	30	36	
PR				
Negative	68	37	31	0.12
Positive	57	26	31	
HER2				
Negative	36	29	7	0.002*
Positive	89	34	55	
Family history of breast cancer				
Yes	73	41	32	0.07
No	52	22	30	
Radiation therapy				
Yes	34	19	15	0.39
No	91	44	47	
Chemotherapy				
Yes	46	22	24	0.31
No	79	41	38	
Menopausal status				
Yes	48	23	25	0.45
No	77	40	37	

*Statistically significant.

ER = estrogen receptor; PR = progesterone receptor; HER-2 = c-erb B-2.

### Correlation of serum DKK-1 levels with OS and RFS

**Figure 2:**
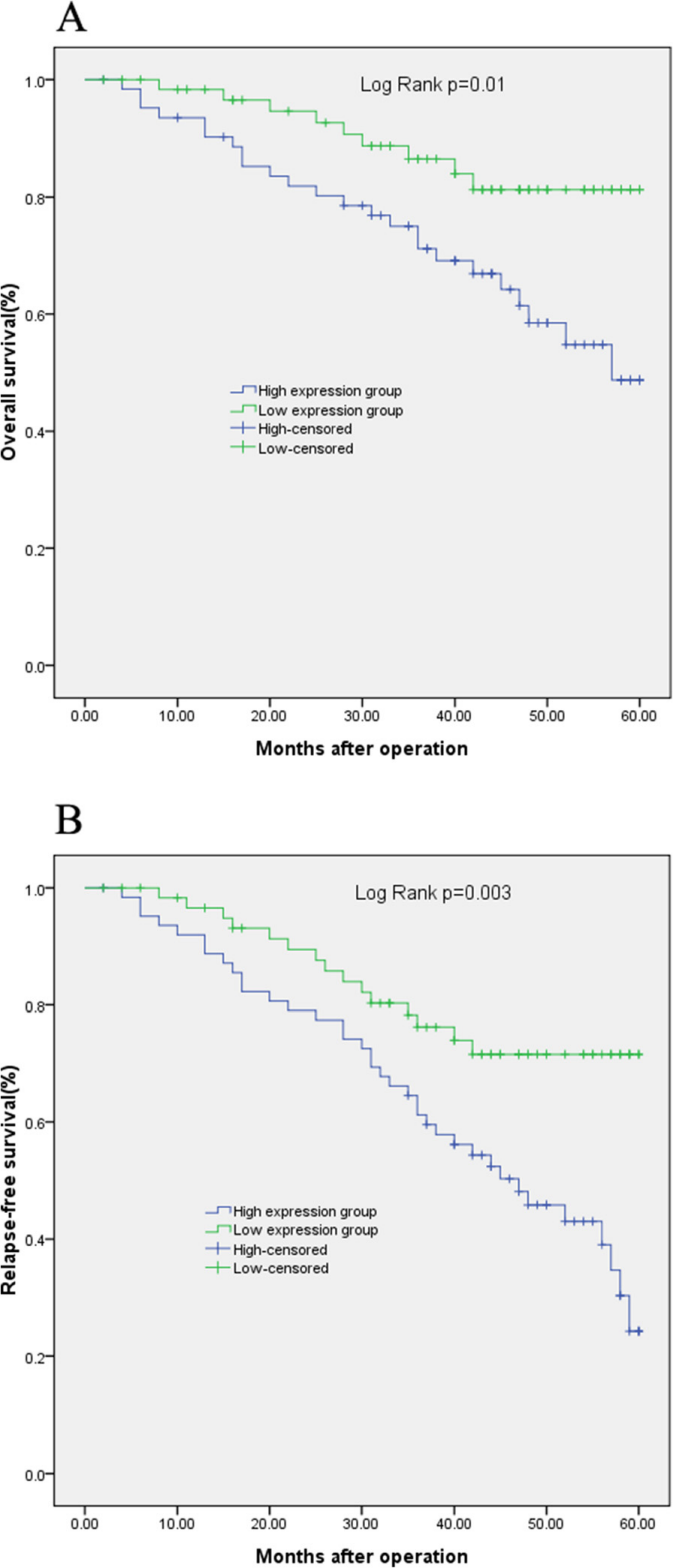
**Survival analysis of the serum DKK-1 levels with overall (A) and relapse-free survival (B) of patients with breast cancer after surgery.**

Considering that serum DKK-1 levels were significantly correlated with TNM stage, tumor grade, and lymph node metastasis, we hypothesized that serum DKK-1 level might affect the prognosis of breast cancer patients. To confirm this possibility, the DKK-1 expression levels and the prognosis of patients with breast cancer were analyzed by using the Kaplan–Meier method. The Kaplan-Meier method for survival analysis showed that the patients with a high serum DKK-1 level had a poorer OS(48.7% vs. 81.3%, *P* = 0.01; Figure 2A) and RFS (24.3% vs. 71.6%, *P* = 0.003; Figure 2B) than those with a low expression level. The multivariate Cox regression analysis indicated that serum DKK-1 level (HR = 2.19; *P* = 0.002), TNM stage (HR = 4.24; *P* = 0.01), and lymph node metastasis (HR = 3.12; *P* = 0.007) were independent prognostic factors for OS, while serum DKK-1 level (HR = 3.97; *P* < 0.001), tumor grade (HR = 2.98; *P* = 0.009), and expression of HER2 (HR = 2.29; *P* = 0.02) were independent prognostic factors for RFS (shown in Table 2).

**Table 2:** **Multivariate analyses for relapse-free survival and overall survival by Cox regression model**

	**Relapse-free survival**			**Overall survival**		
**Variable**	**Hazard ratio**	**95% CI**	**P-value**	**Hazard ratio**	**95% CI**	**P-value**
Age	1.06	0.23-2.35	0.57	1.21	0.35-1.30	0.23
TNM stage	3.29	0.89-7.23	0.06	4.24	3.21-9.22	0.01*
Tumor grade	2.98	1.29-6.77	0.009*	3.83	0.87-4.91	0.08
Histological type	0.81	0.27-3.23	0.62	0.91	0.37-1.29	0.36
Lymph node metastasis	2.21	0.98-8.22	0.07	3.12	2.46-10.92	0.007*
ER	1.28	0.82-2.33	0.29	1.81	0.23-3.11	0.63
PR	1.87	0.28-2.99	0.32	1.34	0.38-3.10	0.29
HER2	2.29	1.88-4.83	0.02*	2.12	0.91-3.91	0.06
DKK-1 expression	3.97	1.82-10.99	<0.001*	2.19	1.28-9.24	0.002*

*Statistically significant.

ER = estrogen receptor; PR = progesterone receptor; HER-2 = c-erb B-2.

## Discussion

Over the past few years, an increasing number of studies have focused on exploring the risk factors of breast cancer in order to prevent it. Up to now, many molecular markers have been used for prognosis of breast cancer patients, including Ki-67, Bcl-2, HER-2, ER, PR, P53, PAR1, and FGFR1 [17-21]. These markers are all correlated with patient outcomes. However, the biological potential of breast cancer is difficult to predict completely with the use of current standard risk factors. Therefore, more persuasive prognostic indicators should be explored for the comprehensive evaluation of breast cancer patients.

DKK-1, DKK-2, DKK-3, and DKK-4, together with a special DKK-3 related protein termed Soggy (Sgy), compose a family of DKK-related genes. DKK-1, DKK-2, DKK-3, and DKK-4 contain 2 discrete cysteine-rich domains, in which the positions of 10 cysteine residues are supremely conserved among family members. DKK-1 and DKK-4, but not DKK-2, DKK-3 or Sgy, have been shown to suppress the Wnt-induced secondary axis induction in Xenopus embryos [22,23]. DKK-4 was found to show high specificity for gastric cancer [24]. DKK-1, which was involved in some aspects of embryonic development, was detected in mature human tissues, mainly in the placenta. Specifically, Wnt-1 protein binds to the frizzled receptor and the low-density lipoprotein receptor-related protein-5/6, triggering signals important for proliferation via β-catenin. DKK-1 binds to low-density lipoprotein receptor-related protein-5/6 and blocks interaction with Wnt-1 resulting in β-catenin degradation and effects on proliferation [25]. Other result showed that DKK-1 functioned not only as an antagonist of the Wnt/β-catenin pathway but also as an agent that could up-regulate other Wnt signaling pathways if the requisite Wnt/receptor combinations were available. DKK-1 also can suppress cell growth and induces apoptotic cell death by activating the c-Jun N-terminal kinase pathway [26]. The expression and roles of DKK-1 are different in various cancers, current studies have reported that overexpression of DKK-1 is found in many malignant tumors, including lung cancer, esophageal carcinomas, cervical cancer, and HCC, indicating a potential oncogenic function of DKK-1 [10-13]. However, paradoxically, the expression of DKK-1 was down-regulated significantly in human colon cancer, gastric cancer and melanoma [14-16]. Therefore, the different function of DKK-1 in different cancer types depends on the histological type of the cancer cells and the tissue microenvironment. However, to our knowledge the expression of DKK-1 in breast cancer is little known. In the present study we investigated the expression of DKK-1 in patients’ serum to establish if DKK-1 can be used as a novel prognostic biomarker in human breast cancer. In this study, we had three findings. Firstly, the serum levels of DKK-1 in patients with breast cancer was significantly higher than that in healthy individuals, and high serum levels of DKK-1 was found to significantly correlate with TNM stage, tumor grade, lymph node metastasis, and expression of HER2. Secondly, Kaplan–Meier analysis showed that breast cancer patients with high serum DKK-1 expression level had distinctly shorter overall survival and relapse-free survival. Thirdly, univariate and multivariate analyses showed that the serum DKK-1 level was independent prognostic parameter of overall survival and relapse-free survival in breast cancer patients. All the results suggested that serum DKK-1 level was befitting to predict prognosis of breast cancer patients after surgery.

## Conclusions

In conclusion, our results indicated that serum DKK-1 was overexpressed in breast cancer and high expression of DKK-1 was associated with poor prognosis. The data suggest that serum DKK-1 may be a useful molecular marker in breast cancer.

## References

[1] Jemal A, Bray F, Center MM, Ferlay J, Ward E, Forman D (2011). Global cancer statistics. CA: Cancer J Clinicians.

[2] Yilmaz M, Christofori G (2010). Mechanisms of motility in metastasizing cells. Mol Cancer Res.

[3] Youssef NS, Hakim SA (2014). Association of Fascin and matrix metalloproteinase-9 expression with poor prognostic parameters in breast carcinoma of Egyptian women. Diagn Pathol.

[4] Wang S, Li H, Wang J, Wang D (2013). Expression of microRNA-497 and its prognostic significance in human breast cancer. Diagn Pathol.

[5] Cheng H, Qin Y, Fan H, Su P, Zhang X, Zhang H, Zhou G (2013). Overexpression of CARM1 in breast cancer is correlated with poorly characterized clinicopathologic parameters and molecular subtypes. Diagn Pathol.

[6] Mao B, Wu W, Davidson G, Marhold J, Li M, Mechler BM, Delius H, Hoppe D, Stannek P, Walter C, Glinka A, Niehrs C (2002). Kremen proteins are Dickkopf receptors that regulate Wnt/beta-catenin signalling. Nature.

[7] Gregory CA, Singh H, Perry AS, Prockop DJ (2003). The Wnt signaling inhibitor dickkopf-1 is required for reentry into the cell cycle of human adult stem cells from bone marrow. J Biol Chem.

[8] Mikheev AM, Mikheeva SA, Liu B, Cohen P, Zarbl H (2004). A functional genomics approach for the identification of putative tumor suppressor genes: Dickkopf-1 as suppressor of HeLa cell transformation. Carcinogenesis.

[9] You L, He B, Uematsu K, Xu Z, Mazieres J, Lee A, McCormick F, Jablons DM (2004). Inhibition of Wnt-1 signaling induces apoptosis in beta-catenin-deficient mesothelioma cells. Cancer Res.

[10] Dong LL, Qu LY, Chu LY, Zhang XH, Liu YH (2014). Serum level of DKK-1 and its prognostic potential in non-small cell lung cancer. Diagn Pathol.

[11] Jiang T, Huang L, Zhang S (2013). DKK-1 in serum as a clinical and prognostic factor in patients with cervical cancer. Int J Biol Markers.

[12] Darlavoix T, Seelentag W, Yan P, Bachmann A, Bosman FT (2009). Altered expression of CD44 and DKK1 in the progression of Barrett’s esophagus to esophageal adenocarcinoma. Virchows Arch.

[13] Wirths O, Waha A, Weggen S, Schirmacher P, Kuhne T, Goodyer CG, Albrecht S, Von Schweinitz D, Pietsch T (2003). Overexpression of human Dickkopf-1, an antagonist of wingless/WNT signaling, in human hepatoblastomas and Wilms’ tumors. Lab Invest.

[14] Gomceli I, Bostanci EB, Ozer I, Kemik AS, Turhan N, Tez M, Kilic S, Demiriz B, Akoglu M (2012). A novel screening biomarker in gastric cancer: serum Dickkopf-1. Hepato-Gastroenterology.

[15] Feldmann R, Schierl M, Fink AM, Sator PG, Maiweg J, Steiner A (2011). Serum levels of glycoprotein Dickkopf-1 in patients with cutaneous malignant melanoma: a prospective pilot study. Dermatology.

[16] Gonzalez-Sancho JM, Aguilera O, Garcia JM, Pendas-Franco N, Pena C, Cal S, de Garcia Herreros A, Bonilla F, Munoz A (2005). The Wnt antagonist DICKKOPF-1 gene is a downstream target of beta-catenin/TCF and is downregulated in human colon cancer. Oncogene.

[17] Smerage JB, Budd GT, Doyle GV, Brown M, Paoletti C, Muniz M, Miller MC, Repollet MI, Chianese DA, Connelly MC, Terstappen LW, Hayes DF (2013). Monitoring apoptosis and Bcl-2 on circulating tumor cells in patients with metastatic breast cancer. Mol Oncol.

[18] Reyal F, Hajage D, Savignoni A, Feron JG, Bollet MA, Kirova Y, Fourquet A, Pierga JY, Cottu P, Dieras V, Fourchotte V, Laki F, Alran S, Asselain B, Vincent-Salomon A, Sigal-Zafrani B, Sastre-Garau X (2013). Long-term prognostic performance of Ki67 rate in early stage, pT1-pT2, pN0, invasive breast carcinoma. PLoS One.

[19] Fokas E, Henzel M, Hamm K, Grund S, Engenhart-Cabillic R (2012). Brain metastases in breast cancer: analysis of the role of HER2 status and treatment in the outcome of 94 patients. Tumori.

[20] Kobayashi T, Iwaya K, Moriya T, Yamasaki T, Tsuda H, Yamamoto J, Matsubara O (2013). A simple immunohistochemical panel comprising 2 conventional markers, Ki67 and p53, is a powerful tool for predicting patient outcome in luminal-type breast cancer. BMC Clin Pathol.

[21] Tiburcio M, Costa SM (2012). M DEFD, Schmitt FC, Longatto Filho A: Characterization of PAR1 and FGFR1 expression in invasive breast carcinomas: Prognostic significance. Oncol Letters.

[22] Yamabuki T, Takano A, Hayama S, Ishikawa N, Kato T, Miyamoto M, Ito T, Ito H, Miyagi Y, Nakayama H, Fujita M, Hosokawa M, Tsuchiya E, Kohno N, Kondo S, Nakamura Y, Daigo Y (2007). Dikkopf-1 as a novel serologic and prognostic biomarker for lung and esophageal carcinomas. Cancer Res.

[23] Krupnik VE, Sharp JD, Jiang C, Robison K, Chickering TW, Amaravadi L, Brown DE, Guyot D, Mays G, Leiby K, Chang B, Duong T, Goodearl AD, Gearing DP, Sokol SY, McCarthy SA (1999). Functional and structural diversity of the human Dickkopf gene family. Gene.

[24] Aung PP, Oue N, Mitani Y, Nakayama H, Yoshida K, Noguchi T, Bosserhoff AK, Yasui W (2006). Systematic search for gastric cancer-specific genes based on SAGE data: melanoma inhibitory activity and matrix metalloproteinase-10 are novel prognostic factors in patients with gastric cancer. Oncogene.

[25] Semenov MV, Tamai K, Brott BK, Kuhl M, Sokol S, He X (2001). Head inducer Dickkopf-1 is a ligand for Wnt coreceptor LRP6. Current Biol.

[26] Polakis P (2000). Wnt signaling and cancer. Genes Dev.

